# Rational Design of A Novel Small-Molecule HIV-1 Inactivator Targeting Both gp120 and gp41 of HIV-1

**DOI:** 10.3389/fphar.2020.613361

**Published:** 2021-01-25

**Authors:** Jing Pu, Yu Dai, Qian Wang, Lu Lu, Junqi Zhang, Wei Xu, Lan Xie, Shengqi Wang, Fei Yu, Xiaoyang He, Shibo Jiang

**Affiliations:** ^1^ Key Laboratory of Medical Molecular Virology of MOE/MOH, School of Basic Medical Sciences & Shanghai Public Health Clinical Center, Fudan University, Shanghai, China; ^2^ Lindsley F. Kimball Research Institute, New York Blood Center, New York, NY, United States; ^3^ Beijing Institute of Radiation Medicine, Beijing, China; ^4^ College of Life Sciences, Hebei Agricultural University, Baoding, China; ^5^ Medical Molecular Virology (MOE/NHC/CAMS), Department of Medical Microbiology and Parasitology, School of Basic Medical Sciences, Shanghai Medical College, Fudan University, Shanghai, China; ^6^ Beijing Institute of Pharmacology and Toxicology, Beijing, China

**Keywords:** HIV-1, entry inhibitor, inactivator, gp120, gp41, Six-helix bundle

## Abstract

Virus inactivator can inactivate cell-free virions without relying on their replication cycle, potentially reducing the impact of viral infection on cells. Previously, we successfully constructed a HIV-1 protein inactivator, 2DLT, by conjugating the D1D2 region of CD4 to the fusion inhibitor T1144 via a 35-amino acid linker. Therefore, it targets both the CD4 binding site in gp120 and NHR region in gp41. Considering that small-molecule agents have the advantages of fast production, low cost, good stability, and oral availability, we herein report the design of a new small-molecule HIV-1 inactivator, FD028, by conjugating FD016 (an analog of NBD-556, a gp120-CD4 binding inhibitor) with FD017 (an analog of 11d, an HIV-1 fusion inhibitor). The results showed that FD028 inactivated cell-free virions at a moderate nanomolar concentration by targeting both HIV-1 gp120 and gp41. Moreover, FD028 has broad-spectrum inhibition and inactivation activity against HIV-1 resistant strains and primary isolates of different subtypes without significant cytotoxicity. Therefore, FD028 has potential for further development as an HIV-1 inactivator-based therapeutic.

## Introduction

Type I human immunodeficiency virus (HIV-1) infection target cells mainly undergo CD4 receptor binding, co-receptor (CXCR4/CCR5) binding, membrane fusion, reverse transcription, DNA integration and protein synthesis (Fanales-Belasio et al., 2010). Inhibitors approved by the U.S. Food and Drug Administration (FDA) for clinical treatment of HIV-1/AIDS (acquired immunodeficiency syndrome) and their combination treatments mainly target the host protein or virus-cell fusion process or the stage after virus entry (https://www.fda.gov). However, inhibitors that target host proteins may affect the normal physiological functions of cells. In addition, reverse transcriptase inhibitors, integrase inhibitors, and protease inhibitors do not block virions from cell entry. Fusion inhibitors acting on the virus-cell fusion stage may have unique advantages in maintaining normal physiological functions of cells and inhibiting viral infection. However, all of these inhibitors rely on the existence of cells to exert their antiviral activity and cannot actively attack cell-free virions. Therefore, the research and development of inactivators that can act independently of the virus replication cycle is very important, albeit not widely studied.

The envelope protein (Env) of HIV-1 consists of two subunits, gp120 and gp41, which mediate viral attachment and membrane fusion. The fusion process is initiated by binding of gp120 to the host CD4 receptor and co-receptor (CXCR4/CCR5), which results in a conformational change in gp41. The fusion peptide (FP) is exposed and inserted into target cells, thus connecting viral and cell membranes. Subsequently, three N-terminal heptad repeats (NHR) and three C-terminal heptad repeats (CHR) fold in an anti-parallel manner to form six-helix bundle (6HB), which shortens the virus-cell distance and induces membrane fusion (Chan et al., 1997; Colman and Lawrence, 2003). Gp120 and gp41 are considered important targets for the development of viral attachment and membrane fusion inhibitors. It has been reported that the first two domains of CD4, D1D2, mimic the binding of CD4 receptor to gp120 and inhibit HIV-1 infection (Berger et al., 1988; Traunecker et al., 1988). On the other hand, T1144, which targets the NHR region of gp41 and inhibits the formation of 6HB, is regarded as the next-generation fusion inhibitor after T20 (Wild et al., 1992; Delmedico et al., 2006). Based on these inhibitors, we previously designed a recombinant protein, 2DLT, by conjugating D1D2 to T1144 through a 35-mer linker (Lu et al., 2012). 2DLT can both target the gp120 CD4 binding site (CD4bs) and the gp41 NHR region, causing cell-free virions to be inactivated at low nanomolar level. Other groups conjugated lectin cyanovirin-N with an MPER peptide to obtain chimeric recombinant protein DAVEI, which simultaneously targets gp120 and gp41, destroying the metastable envelope protein and inactivating the virus (Contarino et al., 2013; Parajuli et al., 2016). However, protein drugs have shortcomings, such as high cost and inconvenient storage and transportation. Compared to proteins and peptides, small-molecule compounds have the advantages of rapid manufacturing, low production cost, good stability and oral absorption. NBD series compounds are promising small molecule entry inhibitors targeting HIV-1 gp120. NBD-556, which was originally identified by Debnath and Jiang's group through screening chemical libraries, can effectively bind to gp120 and block gp120-CD4 binding (Zhao et al., 2005). Subsequently, the crystallographic analysis reveals that NDB-556 targets the Phe43 cavity of gp120 (Kwon et al., 2012). Several analogues of NDB-556 with improved inhibitory activity have been reported (Curreli et al., 2016; Curreli et al., 2017; Curreli et al., 2018). Jiang and colleagues have reported a series of small molecule compounds, such as ADS-J1, NB-2, NB-64 and 11d, with inhibitory activity on the 6HB formation and HIV-1 infection by targeting the NHR of the HIV-1 gp41 (Debnath et al., 1999; Jiang et al., 2004; Wang et al., 2009). Inspired by the design concept of 2DLT and DAVEI, here we conjugated FD017, a new gp41 NHR-targeting compound designed based on the structure of HIV-1 fusion inhibitor 11d (Katritzky et al., 2009) with FD016, an analog of NBD-556 (Narumi et al., 2013) via a basic 2-oxoethylene linker to generate the compound FD028. We then evaluated FD028’s inhibitory activity and inactivation activity against divergent HIV-1 strains and investigated its mechanism of action.

## Materials and Methods

### Chemistry

All chemical reagents were commercially available and used without any further purification unless otherwise mentioned. The reaction process was monitored by analytical TLC (silica gel GF254). Flash column chromatography was carried out with Teledyne Isco Combiflash Rf200 purification. ^1^H-NMR (400 MHz) and ^13^C-NMR (100 MHz) were recorded on a JEOL JNM-ECA-400 spectrometer with tetramethylsilane as internal standard. Mass spectra of molecules were measured on an Agilent 1260-G6230A mass spectrometer.

#### Microwave-assisted Amidation Synthesis of N^1^-(4-Chloro-3-Fluorophenyl)-N^2^-(2,2,6,6-Tetramethylpiperidin-4-yl) Oxalamide (**FD016**)

To a solution of 1 (300 mg, 1.22 mmol) and 2 (191 μL, 1.11 mmol) was added Et_3_N (310 μL, 2.22 mmol), and the reaction mixture was heated in a microwave to 150°C for 3 h. After the reaction reached completion, the resulting mixture was concentrated and purified by flash column chromatography (0-5% MeOH/DCM) to afford the product FD016 as a white solid (355 mg, 90%). ^1^H NMR (400MHz, DMSO-d_6_) *δ* 10.96 (s, 1H), 8.88 (d, 1H, J = 8.1 Hz), 7.96–7.92 (m, 1H), 7.73–7.71 (m, 1H), 7.60 (t, 1H, J = 8.7 Hz), 4.18–4.14 (m, 1H), 1.61 (t, 2H, J = 9.3 Hz), 1.29–1.05 (m, 15H); ^13^C NMR (100MHz, DMSO-d_6_) δ 159.03, 158.62, 157.82, 155.38, 138.15, 138.05, 130.37, 117.08, 114.00, 108.36, 108.10, 50.43, 43.02, 42.43, 34.06, 28.21; ESI-MS (m/z) 356.16 [M + H]^+^.

#### Microwave-assisted Heterocyclization Synthesis of 3-(4-Hydroxyphenethyl)-2-Thioxothiazolidin-4-one (**4**)

To a suspension of 3 (2 g, 14.6 mmol) and bis(carboxymethyl) trisulfide carbonate (3.46 g, 15.3 mmol) in 15 mL i-PrOH was added Et_3_N (3.05 mL, 21.9 mmol), and the reaction mixture was heated in a microwave to 90°C for 45 min. The resulting mixture was slowly poured into 100 mL of ice-water and allowed to stand at room temperature overnight. The precipitate was filtrated out to afford rhodanine 4 as a pale-yellow solid (3.11 g, 84%). ^1^H NMR (400MHz, CDCl_3_) *δ* 7.13 (t, 2H, J = 8.40 Hz), 6.79–6.76 (m, 2H), 4.89 (s, 1H), 4.17–4.13 (m, 2H), 3.93 (s, 2H), 2.89–2.85 (m, 2H). ESI-MS(m/z) 254.01 [M + H]^+^.

#### Knoevenagel Condensation Synthesis of (Z)-2-Chloro-5-(5-((3-(4-Hydroxyphenethyl)-4-oxo-2-Thioxothiazolidin-5-ylidene)methyl)furan-2-yl)benzoic Acid (**FD017**)

To a suspension of 4 (100 mg, 0.4 mmol) and 5 (104 mg, 0.42 mmol) in 5 mL EtOH was added CH_3_COONH_4_ (3 mg, 0.04 mmol), and the reaction was heated to 80°C for 2 h. After the reaction reached completion, the resulting mixture was allowed to naturally cool to room temperature and then filtrated to afford the product FD017 as a red solid (67 mg, 35%). ^1^H NMR (400MHz, DMSO-d_6_) δ 7.93 (d, J = 2.0 Hz, 1H), 7.73 (dd, J = 8.4, 2.0 Hz, 1H), 7.60 (s, 1H), 7.58 (t, 1H, J = 8.4 Hz), 7.36 (dd, J = 10.0, 4.0 Hz, 2H), 7.01 (d, J = 8.4 Hz, 2H), 7.71 (d, J = 8.4 Hz, 2H), 4.16 (t, J = 7.6 Hz, 2H), 2.84 (t, J = 7.6 Hz, 2H); ^13^C NMR (100MHz, DMSO-d_6_) *δ* 193.55, 166.30, 156.87, 156.11, 149.42, 130.72, 129.59, 127.53, 127.01, 124.93, 123.18, 118.63, 115.37, 110.89, 45.61, 31.36; ESI-HRMS (m/z) 486.0223 [M + H]^+^.

#### Selectively Primary Amino Protection Synthesis of 2,2,6,6-Tetramethyl-N-tritylpiperidin-4-amine (**7**)

To a solution of 2 (3.4 mL, 19.8 mmol) and pyridine (5.1 mL, 63.3 mmol) in 30 mL CH_3_CN was slowly added TrCl (5.9 g, 21.1 mmol) at room temperature for 2 h. After the reaction reached completion, a large amount of white solid was precipitated out, filtered out, and then dried under vacuum to afford product 7 as a white solid (7.34 g, 93%). ^1^H NMR (400MHz, DMSO-d_6_) *δ* 9.08 (br s, 1H), 7.76 (br s, 1H), 7.51–7.18 (m, 15H), 2.70 (m, 1H), 1.27–1.19 (m, 8H), 1.03–0.98 (m, 8H).

#### Amidation Synthesis of 2-Chloro-1-(2,2,6,6-Tetramethyl-4-(tritylamino)piperidin-1-yl)ethanone (**8**)

To a solution of 7 (2.4 g, 6.02 mmol) and Et_3_N (2.52 mL, 18.06 mmol) in 20 mL DCM was dropwise added chloroacetyl chloride (560 μL, 7.43 mmol) at 0°C. The reaction was allowed to stir at room temperature for 2 h. After the reaction reached completion, the mixture was extracted using DCM and brine, and the organic phases were collected and dried using MgSO_4_ for final filtration and concentration. The resulting residue was purified by flash column chromatography (0-5% MeOH/DCM) to afford product 8 as a white solid (1.38 g, 48%). ^1^H NMR (400MHz, CDCl_3_) δ 7.55–7.26 (m, 15H), 4.03 (s, 2H), 2.95–2.91 (m, 1H), 1.64–1.58 (m, 2H), 1.45–1.36 (m, 8H), 1.20 (s, 6H).

#### Nucleophilic Substitution Synthesis of Tert-butyl 4-(2-oxo-2-(2,2,6,6-tetramethyl-4-(tritylamino)piperidin-1-yl)ethoxy)phenethylcarbamate (**9**)

A solution of 8 (1.5 g, 3.2 mmol) was prepared in 15 mL DMF, and to the solution were successively added K_2_CO_3_ (869 mg, 6.3 mmol), TBAI (155 mg, 0.21 mmol) and N-Boc-tyramine (898 mg, 3.78 mmol). The reaction mixture was heated to 80°C for 5 h. After the reaction reached completion, the mixture was extracted using EtOAc, and the organic phases were collected, washed with brine, and dried over Na_2_SO_4_ for final filtration and concentration. The resulting residue was purified by flash column chromatography (0-50% EtOAc/PE) to afford product 9 as a yellow oil (1.65 g, 77%). ^1^H NMR (400MHz, CDCl_3_) *δ* 7.56–7.18 (m, 15H), 7.07–6.79 (m, 4H), 4.55 (s, 2H), 3.32–3.30 (m, 2H), 2.95 (p, J = 7.2 Hz, 1H), 2.75–2.68 (m, 2H), 1.47–1.34 (m, 19H), 1.22 (s, 6H).

#### Acidolysis of Trityl Synthesis of Tert-butyl 4-(2-(4-amino-2,2,6,6-tetramethylpiperidin-1-yl)-2-oxoethoxy)phenethylcarbamate (**10**)

Compound 9 (1 g, 1.48 mmol) was added to 10 mL 50% AcOH aqueous solution, and the reaction mixture was stirred at room temperature for 0.5 h. After the reaction reached completion, the mixture was neutralized using saturated Na_2_CO_3_ solution and extracted by EtOAc. The organic phases were collected, washed with brine, and dried over Na_2_SO_4_ for final filtration and concentration. The resulting residue was purified by flash column chromatography (0–15% MeOH/DCM) to afford product 10 as a white solid (480 mg, 75%). ^1^H NMR (400MHz, DMSO-d_6_) *δ* 7.09 (d, 2H, J = 8.8 Hz), 8.50 (br s, 1H), 7.88 (d, J = 8.8 Hz, 2H), 4.65 (s, 2H), 3.42–3.34 (m, 1H), 3.10–3.05 (m, 2H), 2.62 (t, J = 8.0 Hz, 2H), 2.02 (dd, J = 14.0, 6.4 Hz, 2H), 1.71 (dd, J = 14.0, 9.0 Hz, 2H), 1.50 (s, 6H), 1.40 (s, 6H), 1.36 (s, 9H); ESI-MS(m/z) 434.30 [M + H]^+^.

#### Amidation Synthesis of Tert-butyl 4-(2-(4-(2-((4-Chloro-3-fluorophenyl)amino)-2-oxoacetamido)-2,2,6,6-Tetramethylpiperidin-1-yl)-2-oxoethoxy)phenethylcarbamate (**11**)

To a solution of 10 (100 mg, 0.23 mmol) in 5 mL toluene were added 1 (50 mg, 0.20 mmol) and Et_3_N (56 μL, 0.40 mmol). The reaction mixture was heated in a microwave to 150°C for 3 h. After the reaction reached completion, the resulting mixture was concentrated to remove the solvents and then purified by flash column chromatography (0–5% MeOH/DCM) to afford product 11 as a white solid (110 mg, 87%). ^1^H NMR (400MHz, CDCl_3_) δ 9.31 (s, 1H), 7.73 (dd, J = 10.6, 2.5 Hz, 1H), 7.54 (d, J = 7.8 Hz, 1H), 7.40 (t, J = 7.8 Hz, 1H), 7.26–7.24 (m, 1H), 7.11 (d, J = 8.4 Hz, 2H), 6.87 (d, J = 8.4 Hz, 2H), 4.65 (s, 2H), 4.53 (br s, 1H), 4.38 (p, J = 7.6 Hz, 1H), 3.35–3.33 (m, 2H), 2.75–2.71 (m, 2H), 2.27 (dd, J = 7.8, 14.6 Hz, 2H), 1.85 (dd, J = 7.8, 14.6 Hz, 2H), 1.61 (s, 6H), 1.55 (s, 6H), 1.43 (s, 9H); ESI-HRMS (m/z) 655.2669 [M + Na]^+^.

#### Tandem Acidolysis of Boc/Microwave-assisted Heterocyclization Synthesis of N^1^-(4-Chloro-3-Fluorophenyl)-N^2^-(2,2,6,6-Tetramethyl-1-(2-(4-(2-(4-oxo-2-Thioxothiazolidin-3-yl)ethyl)phenoxy)acetyl)piperidin-4-yl)oxalamide (**6**)

To a suspension of 11 (700 mg, 1.1 mmol) in 6 mL MeOH was slowly added AcCl (0.24 mL, 3.4 mmol) at 0°C, and the reaction was allowed to warm to room temperature and then stirred for 1 day. The reaction mixture was concentrated to remove the solvents, and after adding 15 mL of ether, the amine hydrochloride intermediate was precipitated. After filtration, the amine hydrochloride intermediate was directly added into a suspension of bis(carboxymethyl) trisulfide carbonate (260 mg, 1.15 mmol) and Et_3_N (305 μL, 2.19 mmol) in 5 mL i-PrOH. The reaction mixture was heated in a microwave to 90°C for 45 min. After the reaction reached completion, the mixture was extracted using EtOAc, and the organic phases were collected, washed with brine, and dried over Na_2_SO_4_ for final filtration and concentration. The resulting residue was purified by flash column chromatography (0–5% MeOH/DCM) to afford rhodanine 6 as a pale-yellow solid (220 mg, 31%). ^1^H NMR (400MHz, CDCl_3_) δ 9.15 (s, 1H), 7.51 (d, J = 9.0 Hz, 1H), 7.43 (dd, J = 10.6, 2.5 Hz, 1H), 7.24 (t, J = 7.8 Hz, 1H), 7.04–6.94 (m, 3H), 6.76 (d, J = 8.8 Hz, 2H), 5.13 (d, J = 9.0 Hz, 1H), 4.83–4.81 (m, 1H), 4.28 (dd, J = 27.4, 14.6 Hz, 2H), 4.09–4.02 (m, 2H), 3.94 (s, 2H), 2.79–2.72 (m, 2H), 1.74–1.65 (m, 8H), 1.47–1.41 (m, 8H); ESI-HRMS (m/z) 649.1713 [M + H]^+^.

#### Knoevenagel Condensation Synthesis of (Z)-2-Chloro-5-(5-((3-(4-(2-(4-(2-((4-Chloro-3-Fluorophenyl)amino)-2-oxoacetamido)-2,2,6,6-Tetramethylpiperidin-1-yl)-2-oxoethoxy)phenethyl)-4-oxo-2-Thioxothiazolidin-5-ylidene)methyl)furan-2-yl)benzoic Acid (**FD028**)

To a suspension of 6 (100 mg, 0.15 mmol) and 5 (42 mg, 0.17 mmol) in 5 mL EtOH was added CH_3_COONH_4_ (1.2 mg, 0.015 mmol), and the reaction mixture was heated to 80°C for 2 h. After the reaction reached completion, the resulting mixture was allowed to naturally cool to room temperature and then filtrated to afford the product FD028 as a red solid (67 mg, 49%). ^1^H NMR (400MHz, DMSO-d_6_) δ 10.95 (s, 1H), 9.02 (d, J = 9.0 Hz, 0.70 H), 8.80 (d, J = 9.5 Hz, 0.32H), 8.12 (s, 0.78 H), 7.88–7.81 (m, 2H), 7.70–7.63 (m, 3H), 7.53 (t, J = 8.7 Hz, 1H), 7.45 (d, J = 3.6 Hz, 1H), 7.38 (d, J = 3.6 Hz, 1H), 7.25–7.23 (m, 1.1H), 7.08–7.02 (m, 2.3 H), 6.84–6.79 (m, 2.4 H), 5.20 (d, J = 9.2 Hz, 0.8H), 4.74–4.65 (m, 1.5H), 4.35–4.29 (m, 2.2H), 4.17–4.11 (m, 2.3H), 2.86–2.80 (m, 2H), 2.33–2.24 (m, 0.42H), 2.17–2.10 (m, 1.5H), 1.90–1.80 (m, 1.1H), 1.68–1.61 (m, 6H), 1.30–1.28 (m, 7H); ESI-HRMS(m/z) 881.1624 [M + H]^+^.

### Cells and Viruses

CEMx174 517 5.25 M7 cells were kindly provided by Dr C. Cheng-Mayer. CHO-WT cells, MT-2 cells, HIV-1 laboratory-adapted strains, primary isolates and T20-resistant strains, N36 and C34 peptides and all the gp120 CD4bs-targeting mAbs were obtained from the NIH AIDS Reagent Program. T2635-resistant mutation was introduced into the HIV-1 LAI Infectious Molecular Clone (NIH AIDS Reagent Program) to produce T2635-resistant strains (Eggink et al., 2011).

### Inhibition of HIV-1 Infection

The inhibitory activities of compounds alone or in combination against HIV-1 laboratory-adapted strains IIIB (X4) and Bal (R5), HIV-1 primary isolates, and T20-and T2635-resistant mutants were detected as previously described (Jiang et al., 1993). Briefly, 50 μL of different concentrations of compound or PBS were incubated with 50 μL of 100 TCID50 (50% tissue culture infective doses) of HIV-1 live virus for 30 min at 37°C. Then, 100 μL of 3 × 10^4^ cells (MT-2 cells for X4 and X4R5 viruses; CEMx 174 5.25 M7 cells for R5 viruses) were added to each well. After overnight incubation, 150 µL of the supernatant were discarded, and the same volume of fresh medium was replenished. After 3-4 days of incubation at 37°C, 50 μL of supernatant per well were collected, and the virus was lyzed with the same volume of 5% Triton X-100. The p24 antigen was detected by ELISA. IC50 (concentration causing 50% inhibition) and combination index (CI) were calculated using CalcuSyn software kindly provided by Dr T. C. Chou, and the optimum curves were drawn using GraphPad Prism software (GraphPad, San Diego, CA, USA).

### Inactivation of HIV-1 Free Virions

The inactivation activities of the compounds, alone or in combination, against different cell-free HIV-1 virions were measured as previously described (Qi et al., 2017). Briefly, different concentrations of compound or PBS were incubated with 800 TCID50 live virus for 1 h at 4°C. Subsequently, PEG-6000 was added to a final concentration of 3% and incubated for another 1 h at 4°C. The virus was collected by centrifugation of the mixture at 13,000 rpm at 4°C for 30 min. The supernatant was discarded, and the virus was resuspended in 3% PEG-6000 containing 10 mg/mL BSA and centrifuged at 13,000 rpm at 4°C for 30 min. The above procedure was repeated twice to completely remove residual compounds. Finally, the virus particles were resuspended in 100 μL of medium and added to 100 μL of 3 × 10^4^ MT-2 or CEMx 174 5.25 M7 cells. After 3-4 days of incubation at 37°C, 50 μL of supernatant per well were collected and mixed with the same volume of 5% Triton X-100. The p24 antigen was detected by ELISA. EC50 (effective concentration causing 50% inactivation) and combination index (CI) were calculated as described previously (Chou, 2006).

### Interaction With gp120

Flow cytometry was used to detect compounds that competed with gp120 CD4bs-targeting mAbs for binding to gp120. In brief, 100 µL of FD016 at a concentration of 100 µM or PBS was first incubated with CHO-WT cells expressing HIV-1 HXB2 gp120 for 30 min at room temperature, and then gp120 CD4bs-targeting mAbs (5 μg/mL, 100 µL) and FITC-labeled goat anti-human secondary antibody were added successively. After three washes, the cells were analyzed with flow cytometry. ELISA was carried out to detect the activity of compounds that inhibit the binding of soluble CD4 (sCD4) or gp120 CD4bs-targeting mAbs to gp120 as previously described (Si et al., 2004; Curreli et al., 2014). Briefly, for sCD4 and gp120 binding, 50 µL of HIV-1 IIIB gp120 at 0.5 μg/mL was captured on polystyrene plate coated with sheep anti-gp120 antibody D7324 (Aalto Bio Reagents). sCD4 (ImmunoDiagnostics, Inc.) at 200 ng/mL was then added in the presence of an equal volume of different concentrations of compounds or PBS and incubated at 37°C for 1 h. Next, rabbit anti-sCD4 IgG (ImmunoDiagnostics, Inc.) at 250 ng/mL was added and incubated at 37°C for another 1 h, followed by goat anti-rabbit IgG-biotin (Invitrogen), streptavidin-horseradish peroxidase (Invitrogen). For gp120 CD4bs-targeting mAbs and gp120 binding, polystyrene plates were coated with 50 µL of HIV-1 IIIB gp120 (2 μg/mL in 0.1 mol/L sodium bicarbonate buffer (pH 8.6)) at 4°C overnight and then blocked with PBS containing 2% BSA at 37°C for 2 h. Next, gp120 CD4bs-targeting mAbs at 2 μg/mL were added in the presence of an equal volume of FD028 at different concentrations and incubated at 37°C for 1 h. Horseradish peroxidase (HRP)-labeled goat-anti-human IgG (Sigma-Aldrich) was added sequentially. After adding 3,3′,5,5′-tetramethylbenzidine chromogen substrate and sulfuric acid, the absorbance at 450 nm (A450) was recorded using a Microplate Reader (Tecan, Männedorf, Switzerland).

### Inhibition of Six-Helix Bundle Formation

The inhibition of the compounds on 6HB formation between N36 and C34-biotin was assessed with ELISA as previously described (Su et al., 2017). In brief, a 96-well polystyrene plate was coated with a 6HB-specific mAb, NC-1 (5 μg/mL, 50 μL) (Jiang et al., 1998). An inhibitor (compound or C-peptide control) at graded concentrations was preincubated with N36 peptide (2 μM, 50 μL) at 37°C for 1 h. C34-biotin was added with a final concentration of 0.5 μM (100 μL) and incubated at 37°C for another 1 h. Subsequently, 50 μL of the mixture were added to the wells of the NC-1-coated plate, followed by incubation for 1 h. Then, HRP-labeled streptavidin and the substrate were added sequentially. The optical density at 450 nm was measured using a microplate reader (Tecan, Männedorf, Switzerland).

The inhibition of the compounds on 6HB formation between N36 and C34 was further evaluated as described previously (Yu et al., 2014). Briefly, the N36 peptides with a final concentration of 40 μM were incubated with different concentrations of FD028 at 37°C for 30 min, then the mixture was added with C34 peptide (the final concentration was 40 µM) and incubated at 37°C for another 30 min. The sample was mixed with Tris-glycine native sample buffer and then loaded onto the 18% Tris-glycine gel. Gel electrophoresis was carried out with 125 V constant voltage at room temperature for 3 h.

### Cytotoxicity of Compounds

Cell viability was detected following the instructions in the protocol provided in the cell counting kit-8 (CCK-8; Dojindo Molecular Technologies, Gaithersburg, MD, USA) as previously described (Yang et al., 2018). Briefly, MT-2 cells and CEMx174 517 5.25 M7 cells (3 × 10^4^/well) were incubated with compounds at different concentrations at 37°C for 48 h. Then, the supernatant was discarded, and CCK8 diluted 10 times with fresh medium was added (200 μL/well). After 2–6 h of incubation at 37°C, the absorbance at 450 nm was measured using a microplate reader (Tecan, Männedorf, Switzerland). The CC50 (50% cytotoxic concentration) was calculated by use of GraphPad Prism software (GraphPad, San Diego, CA, USA).

## Results

### Synthesis of FD016 and FD017

NBD-556 analog FD016 was synthesized from N-(4-chloro-3-fluorophenyl) oxalamic acid ethyl ester 1 (Narumi et al., 2013) as starting material following the previously reported method (Debnath et al., 2006). Microwave-assisted amidation reaction of ester 1 with 4-amino-2,2,6,6-tetramethylpiperidine 2 in toluene at 150°C for 3 h gave FD016 in 90% yield. For the preparation of gp41-targeting compound FD017, N-substituted rhodanine 4 was first synthesized by a literature procedure (Radi et al., 2010a), and tyramine 3 was treated with bis(carboxymethyl) trithiocarbonate in i-PrOH at 90°C in a microwave for 45 min to give 4 in 84% yield. Subsequently, Knoevenagel condensation of 4 with aldehyde 5 in EtOH in the presence of a catalytic amount of ammonium acetate at reflux temperature for 2 h afforded FD017 in 35% yield.

**Scheme 1:**
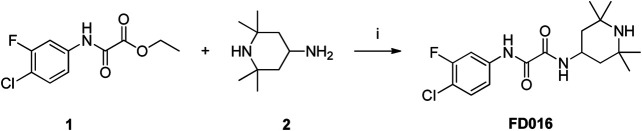
Synthesis of FD016. Reagents and conditions: (i) Et_3_N, Toluene, MW, 150°C, 3 h.

**Scheme 2:**
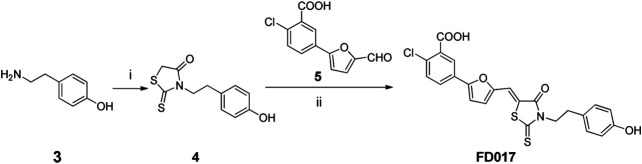
Synthesis of FD017. Reagents and conditions: (i) bis(carboxymethyl) trithiocarbonate, Et_3_N, i-PrOH, MW, 90°C, 45 min; (ii) CH_3_CO_2_NH_4_, EtOH, reflux, 2 h.

### Detection of HIV-1 Inhibition and Inactivation Activity of FD016 and FD017

**FIGURE 1:**
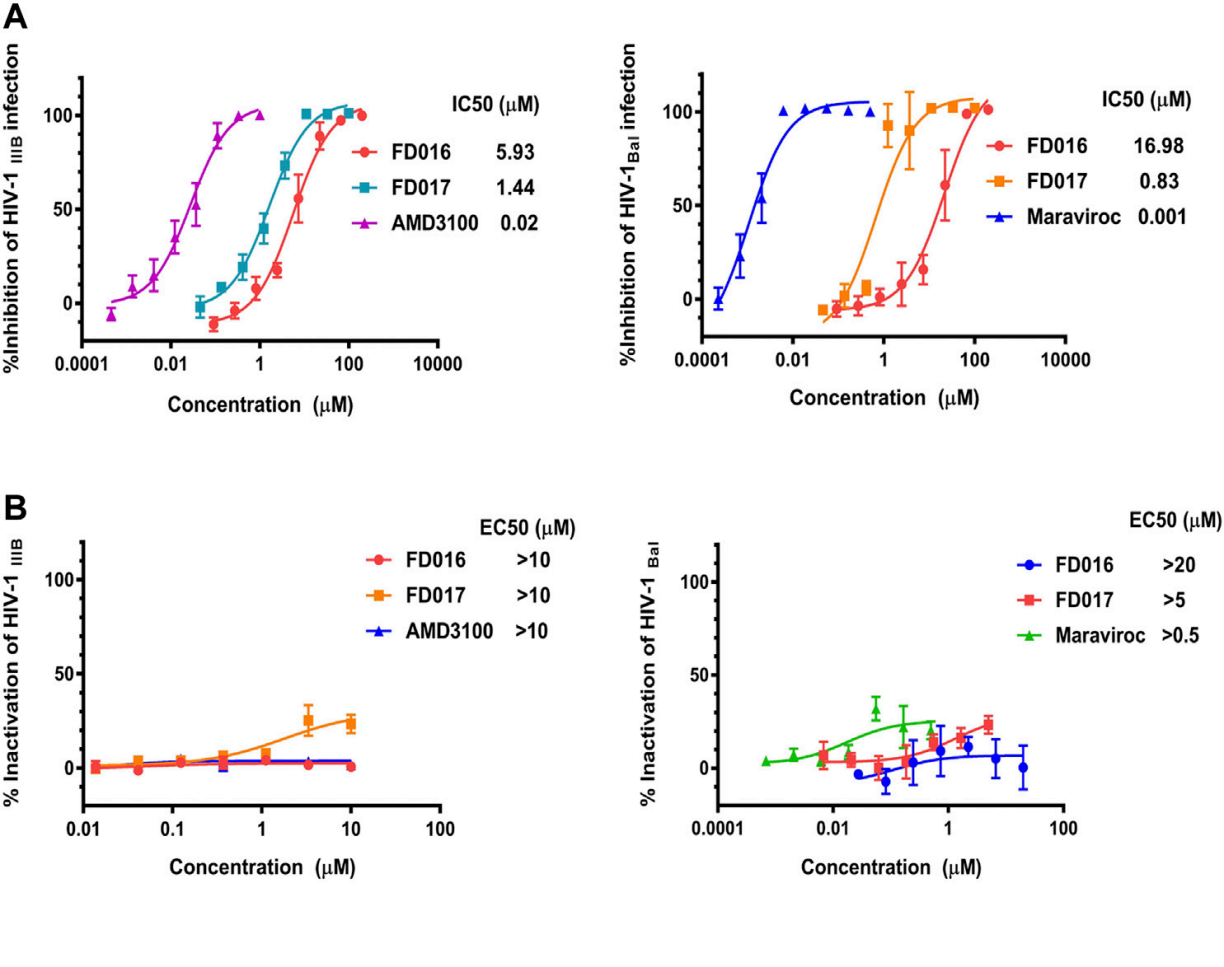
Inhibition and inactivation activity of FD016 and FD017 on HIV-1 laboratory-adapted strains. **(A)** Inhibitory activity against IIIB and Bal infection. **(B)** Inactivation of cell-free IIIB and Bal. Each experiment was repeated twice, and similar results were obtained. Data are means ± SD of triplicate samples from a representative experiment.

First, we measured the activity of FD016 in inhibiting HIV-1 laboratory-adapted strains IIIB and Bal, using AMD3100, a CXCR4 antagonist, and Maraviroc, a CCR5 antagonist, as positive controls for inhibition of IIIB and Bal infection, respectively. Consistent with previous reports, FD016 is effective in inhibiting HIV-1 infection with IC50 of 5.93 and 16.98 μM for IIIB and Bal, respectively (Figure 1A) (Narumi et al., 2013). Our newly designed compound, FD017, could also inhibit HIV-1 infection (IC50 = 1.44 and 0.83 μM for IIIB and Bal, respectively) (Figure 1A). Although many inhibitors against HIV-1 have been reported, most cannot inactivate the virus. Thus, we separated the virus and compound using the previously reported PEG precipitation method to detect whether FD016 and FD017 did, indeed, have inactivation activity (Qi et al., 2017). The compounds were incubated with the virus at 4°C, the virus was precipitated by PEG-6000, and the compounds were washed away. The obtained virus was then used to infect the target cells to detect their post-incubation infectivity. As shown in Figure 1B, the virus was able to infect target cells, even after treatment with FD016 or FD017 at a concentration up to 10 μM (EC50 > 10 μM). This indicates that neither FD016 nor FD017 could inactivate cell-free HIV-1. In short, the above results demonstrated that both FD016 and FD017 had inhibitory activity against HIV-1, but neither one could inactivate cell-free virions.

### Study of the Mechanism of Action of FD016 and FD017 by Targeting gp120 and Blocking 6HB Formation

**FIGURE 2:**
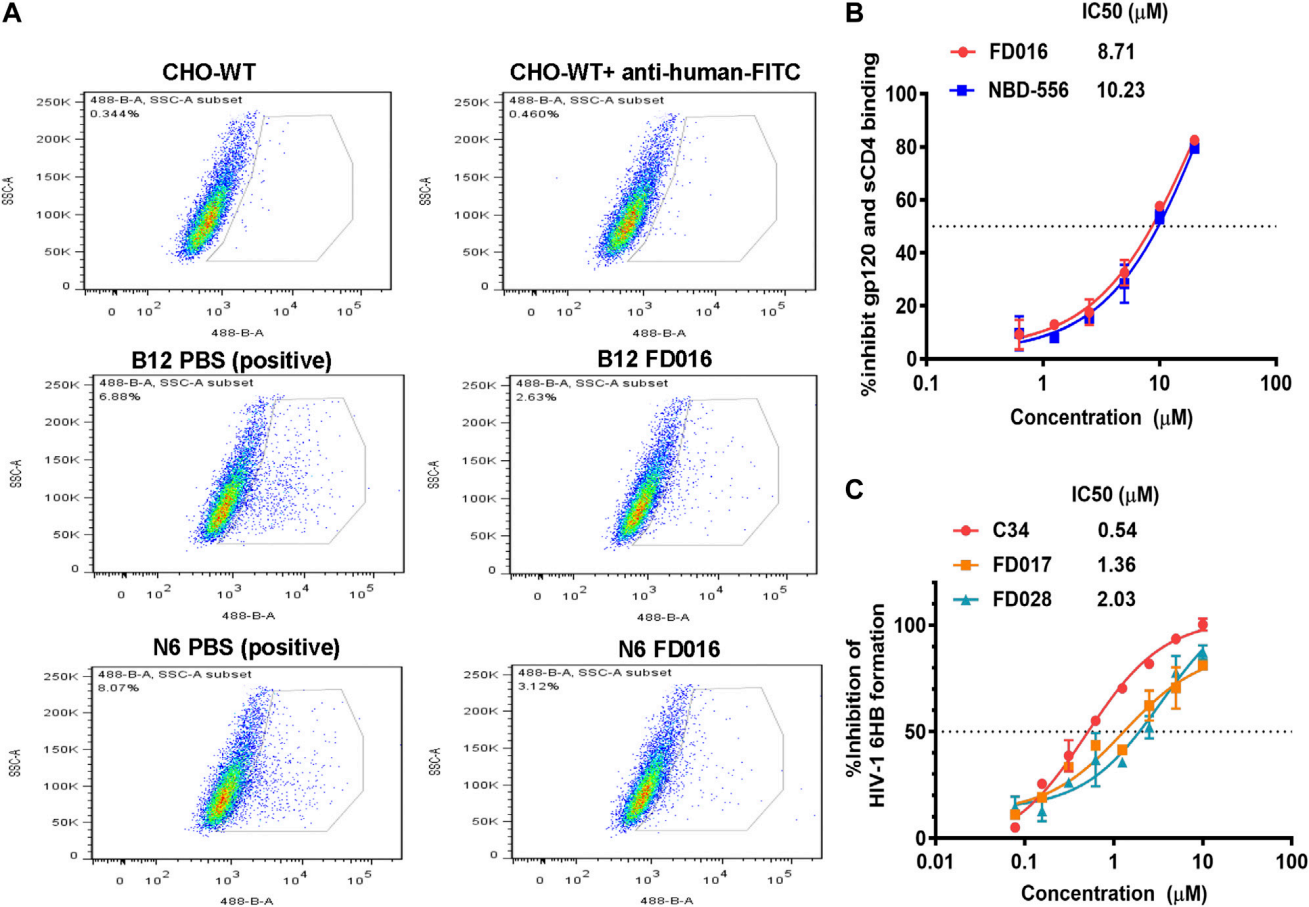
The mechanism of action of FD016 and FD017. **(A)** FD016 competitively inhibits the binding of B12/N6 to gp120 at a concentration of 100 µM. Fluorescein isothiocyanate (FITC)-conjugated goat anti-human IgG was used to detect the presence of B12/N6. **(B)** The inhibitory activity of FD016 and NBD-556 on the binding of gp120 and sCD4. **(C)** Inhibitory activities of C34, FD017 and FD028 against 6HB formation between N36- and C34-biotin peptides. The final concentration of N36- and C34-biotin peptides was 0.5 μM. Each experiment was repeated twice, and similar results were obtained. Data are means ± SD of triplicate samples from a representative experiment.

FD016 is an analog of NBD-556, which targets the CD4bs of gp120 (Narumi et al., 2013) (Kwon et al., 2012; Zhao et al., 2005). Therefore, we first tested the inhibitory activity of FD016 on gp120 CD4bs mAbs (B12 and N6) binding to HIV-1 HXB2 gp120 expressing CHO cells (CHO-WT) by flow cytometry. The result showed that FD016 could inhibit the interaction of gp120 and CD4bs-targeting mAbs effectively at a concentration of 100 µM (Figure 2A). Furthermore, we directly measured the effect of FD016 on the binding of gp120 to sCD4 by ELISA. As shown in Figure 2B, the IC50 of FD016 to inhibit this interaction is 8.71 µM, which is similar to the activity of NBD-556 (Curreli et al., 2014). Then, our newly designed gp41-targeting compound, FD017, was tested for its inhibitory effect on 6HB formation. As shown in Figure 2C, FD017 inhibited the formation of 6HB between N36 and C34-biotin peptides in a dose-dependent manner with an IC50 of 1.36 µM. The above results indicate that FD016 and FD017 can interact with gp120 and gp41, respectively, indicating their inhibitory efficacy.

### HIV-1 Inhibition and Inactivation Activity of the FD016/FD017 Combination

Adherence to combination therapy can effectively control the viral load of HIV-1 (Chesney et al., 2000). Some studies have shown that the combination of inhibitors of different targets can increase the inhibition of HIV-1, may have a higher genetic barrier, and may delay the production of resistant strains (Mathys and Balzarini, 2014; Titanji et al., 2017). Since FD016 and FD017 target gp120 and gp41, respectively, we combined them proportionally based on their IC50 values and tested whether they had synergistic effect in inhibiting and inactivating HIV-1. The results showed that the combination of FD016 and FD017 exhibited synergistic effect against HIV-1 IIIB and Bal infection with CI values of 0.60 and 0.71, respectively (Table 1). Meeting our expectations, the combination of the two compounds could also inactivate cell-free HIV-1 virions (Table 1).

**TABLE 1:** HIV-1 inhibition and inactivation activity of the FD016 and FD017 combination.

Virus	^a^IC50 of FD016 (μM)		Dose reduction (fold)	^a^IC50 of FD017 (μM)		Dose reduction (fold)	^c^CI
	Alone	In mixture		Alone	In mixture		
IIIB	4.40	1.19	3.69	1.80	0.59	3.05	0.60
Bal	18.76	1.82	10.31	1.48	0.91	1.63	0.71

Virus	^b^EC50 of FD016 (μM)		Dose reduction (fold)	^b^EC50 of FD017 (μM)		Dose reduction (fold)	
	Alone	In mixture		Alone	In mixture		
IIIB	>10	2.51	>3.98	>10	1.21	>8.26	—
Bal	>20	2.63	>7.60	>5	1.38	>3.62	—

^a^ IC50, 50% inhibition of infection *in vitro*.

^b^EC50, 50% effective concentration of virus inactivation.

^c^CI < 1, = 1 and >1 indicate synergism, additive effect, and antagonism, respectively.

### Synthesis of FD028 (FD017-Linker-FD016)

Antiretroviral therapy is usually a combination of multiple drugs (Chesney et al., 2000), and considering the model of action of 2DLT, we therefore used a simple linker to conjugate FD016 and FD017 into a bifunctional compound, which is expected to both inhibit HIV-1, but also inactivate it.

Synthesis of the FD028 conjugate was performed by Knoevenagel condensation of aldehyde 5 with N-substituted rhodanine 6 which comprised the NBD-556 analog FD016. First, amino 2 was selectively protected with TrCl in the presence of pyridine in CH_3_CN for 2 h at room temperature to produce trityl derivative 7 in 93% yield, and amidation of 7 with chloroacetyl chloride gave 8 in 48% yield. Subsequently, compound 8 was treated with N-Boc-tyramine and K_2_CO_3_ in the presence of catalytic amount of TBAI in DMF for 5 h at 80°C to afford 9 in 77% yield. The trityl group of 9 was removed using 50% HOAc aqueous solution for 0.5 h at room temperature to produce 10 in 75% yield. Then, compound 10 was subjected to microwave-assisted amidation reaction using ester 1 in toluene for 3 h at 150°C to give amidate derivative 11 according to the literature procedure (Debnath et al., 2006). The Boc group of 11 was hydrolyzed via treatment with AcCl-MeOH (Nudelman et al., 1998) at room temperature for 1 day, and the resulting amino group was directly reacted with bis(carboxymethyl) trisulfide carbonate under microwave in the presence of Et_3_N in i-PrOH at 90°C for 45 min to afford the highly functionalized rhodanine derivative 6 in 31% yield (Radi et al., 2010b). Finally, the conjugate FD028 was prepared via treatment with 5 and 6 under the same conditions as those of FD017 in 49% yield.

**Scheme 3:**
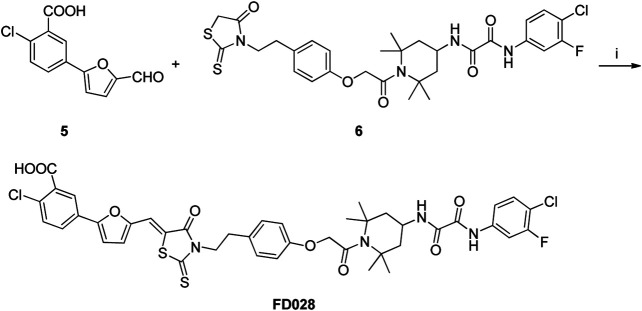
Synthesis of FD028 conjugate. Reagents and conditions: (i) CH_3_CO_2_NH_4_, EtOH, reflux, 2 h.

**Scheme 4:**
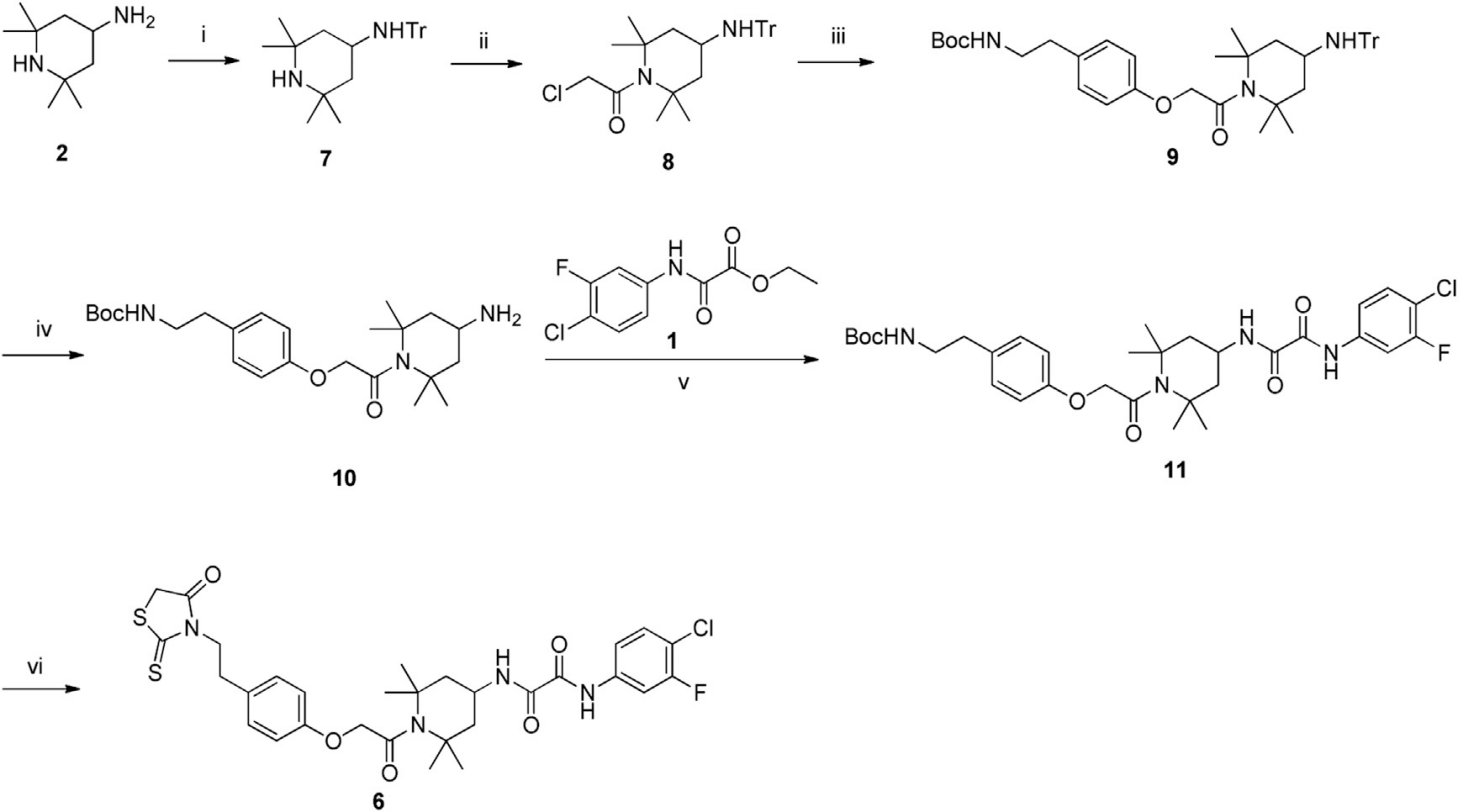
Synthesis of Rhodanine 6. Reagents and conditions: (i) TrCl, Pyridine, CH_3_CN, r.t., 2 h; (ii) chloroacetyl chloride, Et_3_N, CH_2_Cl_2_, 0°C-r.t., 2 h; (iii) tert-butyl 4-hydroxyphenethylcarbamate, K_2_CO_3_, DMF, 80°C, 5 h; (iv) 50% AcOH, r.t., 0.5 h; (v) Et_3_N, Toluene, MW, 150°C, 3 h; (vi) (1) AcCl-MeOH, r.t., 1 h; (2) bis(carboxymethyl) trithiocarbonate, Et_3_N, i-PrOH, MW, 90°C, 45 min.

### Detection of HIV-1 Inhibition and Inactivation Activity of FD028 (FD017-Linker-FD016)

**FIGURE 3:**
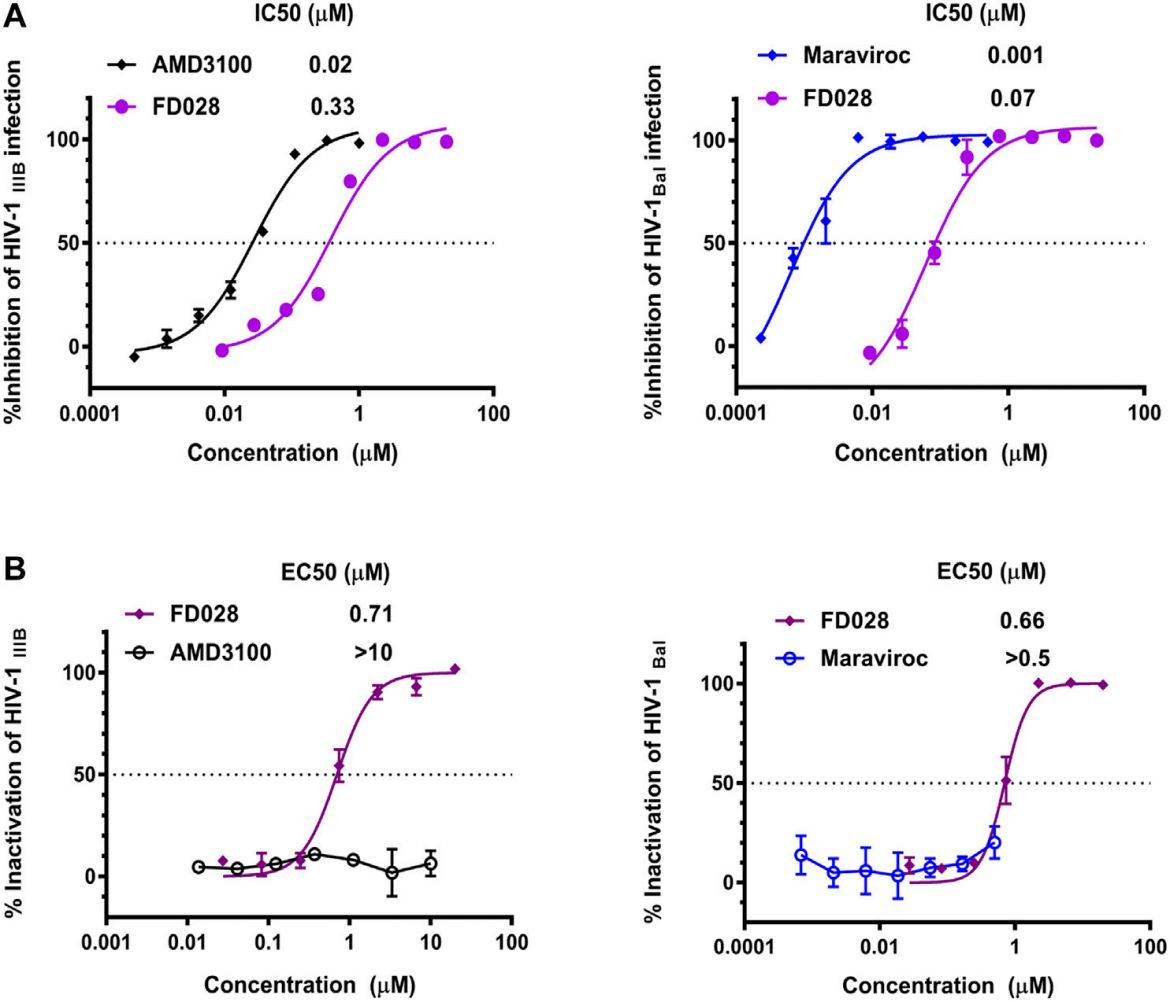
Inhibition and inactivation activity of FD028 on HIV-1 laboratory-adapted strains. **(A)** Inhibitory activity against IIIB and Bal infection. **(B)** Inactivation of cell-free IIIB and Bal. Each experiment was repeated twice, and similar results were obtained. Data are means ± SD of triplicate samples from a representative experiment.

We first tested the inhibition and inactivation activities of the FD028 conjugate on laboratory-adapted strains. The results showed that the average inhibitory activity of FD028 on IIIB and Bal infection was 57-fold and 6-fold of FD016 and FD017, respectively (Figures 1A and 3A). Excitingly, FD028 also showed the ability to inactivate cell-free HIV-1 with EC50 = 0.71 µM for IIIB and 0.66 µM for Bal (Figure 3B). AMD3100 and Maraviroc, which are CXCR4 and CCR5 inhibitors, respectively, could not inactivate virions, even at concentrations up to 500-fold of their IC50 values.

Next, we further evaluated the antiviral activity of FD028 on a variety of clinical strains with different subtypes and different tropisms, as well as drug-resistant strains. The results showed that FD028 had a broad spectrum of inhibition of HIV-1, including T20-resistant viruses and T2635-resistant viruses (Table 2). More importantly, FD028 also showed broad-spectrum inactivation activity of cell-free virions, including resistant viruses (Table 3).

**TABLE 2:** Inhibition of HIV-1 clinical and resistant virus infection.

Virus	^a^ IC50 (μM)	
	AMD3100	FD028
HIV-1 clinical virus		
96USSN20 (A, R5 and X4)	0.11 ± 0.007	0.20 ± 0.01
92/UG/001 (D, R5 and X4)	0.18 ± 0.021	0.45 ± 0.12
92/UG/024 (D, X4)	0.11 ± 0.024	1.04 ± 0.16
93/BR/020 (F, R5 and X4)	0.12 ± 0.031	0.38 ± 0.03
T20-resistant virus		
WT (NL4-3 D36G)	0.02 ± 0.004	0.17 ± 0.06
V38A	0.07 ± 0.023	1.09 ± 0.13
V38A N42T	0.01 ± 0.001	0.95 ± 0.06
V38A N42D	0.04 ± 0.008	0.48 ± 0.11
V38E N42S	0.02 ± 0.004	0.24 ± 0.02
T2635-resistant virus		
WT (LAI)	0.02 ± 0.002	0.45 ± 0.09
K90E	0.02 ± 0.004	0.79 ± 0.28
N113E	0.05 ± 0.004	0.57 ± 0.13
Q66R N126K	0.05 ± 0.017	0.32 ± 0.08
Q79E N126K	0.01 ± 0.002	0.20 ± 0.07
K90E N126K	0.01 ± 0.003	0.46 ± 0.11
H3C	0.004 ± 0.002	0.27 ± 0.06

H3C is a T2635-resistantHIV-1 variant with multiple mutations in gp41, including A6V L33S Q66R N126K H132Q E136G.

^a^ IC50 data were derived from the results of three independent experiments and expressed as means ± SD.

**TABLE 3:** Broad-spectrum inactivating activity of FD028 on cell-free virions.

	^a^EC50 (μM)		
	AMD3100	Maraviroc	FD028
HIV-1 clinical virus			
92/TH/009 (A/E, R5)		>0.5	0.26 ± 0.06
KNH1135 (A, R5)		>0.5	1.23 ± 0.32
SM145/GS 016 (C, R5)		>0.5	0.96 ± 0.09
PBL288 (C, R5)		>0.5	0.54 ± 0.18
T20-resistant virus			
D36G (NL4-3 WT)	>10		0.41 ± 0.02
V38A N42D	>10		0.63 ± 0.14
V38A	>10		0.78 ± 0.19
V38E N42S	>10		0.49 ± 0.03
V38A N42T	>10		0.32 ± 0.11
T2635-resistant virus			
WT (LAI)	>10		0.04 ± 0.01
K90E	>10		0.55 ± 0.23
N113E	>10		1.20 ± 0.16
Q66R N126K	>10		0.14 ± 0.07
Q79E N126K	>10		0.12 ± 0.02
K90E N126K	>10		0.49 ± 0.06
Q66R N113E	>10		0.36 ± 0.03
H3C	>10		1.20 ± 0.14

H3C is a T2635-resistantHIV-1 variant with multiple mutations in gp41, including A6V L33S Q66R N126K H132Q E136G.

^a^ EC50 data were derived from the results of three independent experiments and expressed as means ± SD.

**FIGURE 4:**
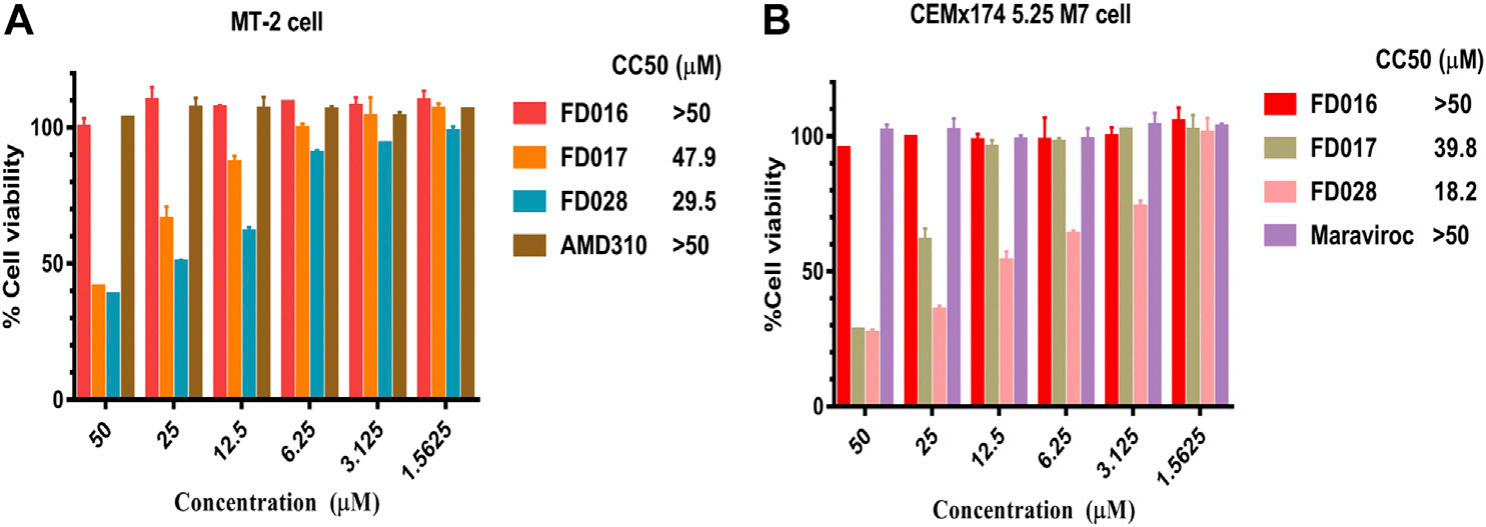
Cytotoxicity of FD028 on target cells. **(A)** Cytotoxicity of compounds on MT-2 cells. **(B)** Cytotoxicity of compounds on CEMx174 5.25 M7 cells. Each experiment was repeated twice, and similar results were obtained. Data are means ± SD of triplicate samples from a representative experiment.

To further evaluate the characteristics of FD028, cytotoxicity on target cells was detected. The results showed that the CC50 of FD028 on MT-2 cells and CEMx174 5.25 M7 cells was 29.5 and 18.2 µM, respectively, which was much higher than the range of inhibitory activity and inactivation activity (Figure 4). These results indicate that we have designed and synthesized a conjugate that is not significantly toxic and has broad-spectrum HIV-1 inhibitory and inactivation activity.

### Study of the Mechanism of Action of FD028 by Blocking 6HB Formation and Targeting gp120

**FIGURE 5:**
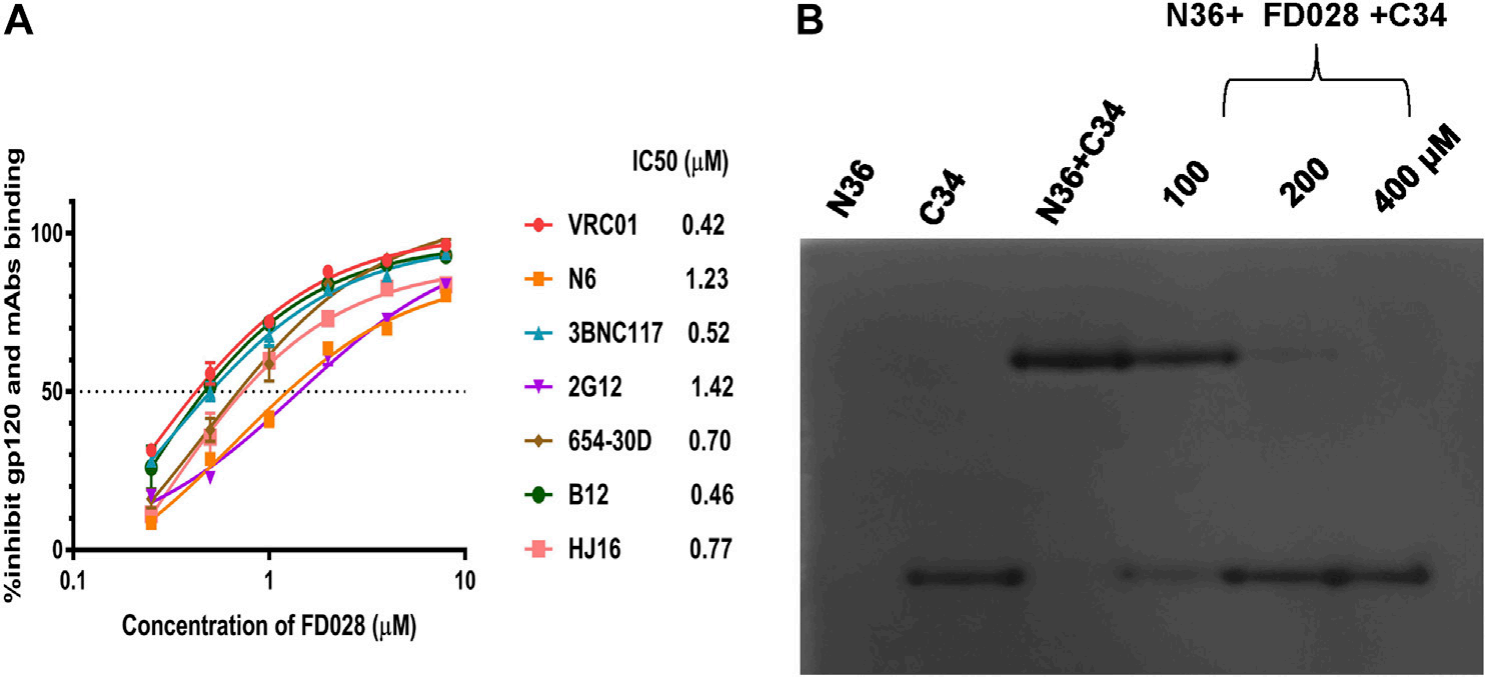
The mechanism of action of FD028. **(A)** ELISA was used to detect that FD028 inhibited the binding of different gp120 CD4bs-targeting mAbs to gp120. VRC01, N6, 3BNC117, 2G12, 654-30D, B12 and HJ16 are anti-HIV-1 gp120 monoclonal antibodies. **(B)** N-PAGE showed that FD028 inhibits 6HB formation between N36 and C34 in a dose-dependent manner. The final concentrations of N36 and C34 peptides were 40 μM. Each experiment was repeated twice, and similar results were obtained. Data are means ± SD of triplicate samples from a representative experiment.

Based on the mechanism of action of the composition, we further evaluated the interaction of FD028 with gp120. As shown in Figure 5A, FD028 could effectively inhibit the binding of gp120 to different gp120 CD4bs-targeting mAbs with IC50 ranging from 0.42 to 1.42 μM. In addition, the results of N-PAGE showed that it inhibited the formation of 6HB in a concentration-dependent manner with IC50 of 100∼200 μM (Figure 5B), while in ELISA, the IC50 value was about of 2 μM (Figure 2C). The discrepancy is possibly because the concentration of N36 and C34 peptides used in N-PAGE is 80-fold higher than that used in ELISA. Studies have shown that the binding of CD4 to gp120 can expose the NHR of gp41 (Si et al., 2004), and FD016 is a CD4 mimic known to induce changes in gp120 conformation (Narumi et al., 2013). Thus, it is possible that FD016 in FD028 binds to gp120 first, leading to conformational changes of Env, and then FD017, which is connected by the linker, binds to the NHR of gp41, eventually leading to the inactivation of virus particles. Therefore, these results confirmed the proof-of-concept design of an inactivator able to target gp120 and gp41.

## Discussion

At present, most anti-HIV-1 drugs used in clinical practice act on the stage after the virus invades the target cells, but without acting upon cell-free virions. However, viral inactivators can act independently of virus-cell fusion by inactivating cell-free virions, thereby protecting the normal cellular function and reducing the risk of the virus forming a latent reservoir after entering cells. In addition, small-molecule compounds are characterized by stable properties, low production costs, and ease of storage. Therefore, inactivators, especially small-molecule inactivators, have unique advantages in treating HIV-1 infection.

**FIGURE 6:**
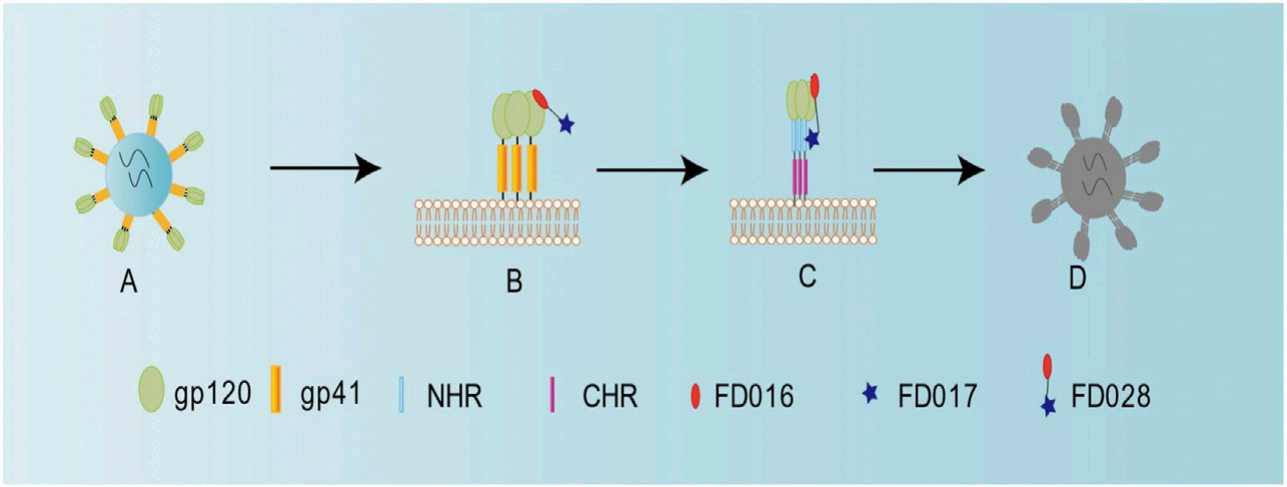
Schematic diagram showing the mechanism of action for FD028 as an inactivator of cell-free HIV-1. **(A)** Cell-free HIV-1 virion. **(B)** FD016 in FD028 binds to gp120. **(C)** The conformation of the envelope protein changes, and FD017 in FD028 binds to the exposed gp41 NHR. **(D)** Cell-free virion loses its infectivity.

Previously, we successfully designed the protein HIV-1 inactivator 2DLT. In this study, we adopted the same idea to design and synthesize the small-molecule HIV-1 inactivator FD028, which represents the FD016-linker-FD017 model. Similar to NBD-556, FD016 targets gp120 and inhibits HIV-1 infection (Figures 1A and 2A,B). Our newly designed FD017 is effective in inhibiting HIV-1 laboratory-adapted strain infection and blocking the formation of 6HB (Figures 1A and 2C). Therefore, the FD028 conjugate not only effectively inhibits HIV-1 infection, but also effectively inactivates cell-free HIV-1, including laboratory-adapted strains, T20-resistant strains, T2635-resistant strains, and primary isolates with different subtypes and tropism (Tables 2, 3). We studied the mechanism of action of FD028 and concluded that it may first bind to gp120 through FD016 to promote the conformational change of Env and then target the NHR of gp41 (Figure 6). In addition, FD028 showed no significant cytotoxicity on target cells MT-2 and CEMx174 5.25 M7 within its activity range (Figure 4).

Although combination ART can usually reduce HIV-1 load to an undetectable level, HIV-1 cannot be cured at present because proviral DNA is integrated into resting memory CD4^+^ T cells in the early stage of acute infection, which is a potential source of virus replication (Wong et al., 1997; Chun et al., 1998). Here, we successfully designed an inactivator that can be used in a shock-and-kill strategy, and it has a broad spectrum of inhibition of HIV-1 infection and HIV-1 inactivation without significant cytotoxicity, making it a good candidate for its development as a HIV-1 inactivator.

## Data Availability Statement

The original contributions presented in the study are included in the article/Supplementary Material, further inquiries can be directed to the corresponding authors.

## Author Contributions

FY, XH, and SJ conceived and designed the experiments. JP, YD, QW, LL, JZ, and WX performed the experiments. JP, LL, LX, and SW wrote the original draft. FY, XH, and SJ reviewed and revised the manuscript.

## Funding

This research was funded by the National Natural Science Foundation of China (81630090 to SJ; 81661128041, 81672019, 81822045 to LL; 81701998 to QW; 81501735 to FY), and the National Science and Technology Major Project of the Ministry of Science and Technology of China (2018ZX10101003-005-010 to JZ)

## Conflict of Interest

The authors declare that the research was conducted in the absence of any commercial or financial relationships that could be construed as a potential conflict of interest.
